# Supplementary material for: Long-term visual field evaluation of trabeculectomy patients with a mean intraocular pressure below 15 mmHg
Source: PLoS One. 2026 Mar 31;21(3):e0345568. doi: 10.1371/journal.pone.0345568 (PMC13037994; doi:10.1371/journal.pone.0345568)
Supplement: Attachment — Submitted filename: Response to Reviewers - 2025-11-20T093348.052.docx [file pone.0345568.s003.docx]

**RESPONSE TO REVIEWERS:**

**Manuscript:** Long-term visual field evaluation of trabeculectomy patients with a mean intraocular pressure below 15 mmHg [PONE-D-25-25183]

**Dear Editor,**

Initially, we would like to thank you and the reviewers for your time and careful review of our manuscript. We believe the editorial process is the best way to enhance the quality of any manuscript.

The responses to all suggestions for our manuscript are described below and highlighted in red in the revised manuscript.

Please let us know if you need any further information.

Sincerely,

**The authors.**

**REVIEWER 1:**

**Covariate control**

The reviewer’s pertinent observation regarding the statistically significant difference in disease severity between groups G1 and G2, defined according to the preoperative MD value, is fully acknowledged. Indeed, group G1 included a significantly higher proportion of eyes with advanced glaucoma, which could represent a potential confounding factor when comparing progression rates between groups.

However, we would like to emphasize that this limitation was considered and controlled in our analyses, as summarized below:

1.	Stratified subanalysis by severity:

Figure 1 shows the correlation between the annual progression rate and mean IOP stratified by severity levels. No significant correlation was observed within any of the strata, in either group G1 or G2, indicating that disease severity did not modify the relationship between IOP and progression.

2.	Interaction analysis between group and severity:

In Figure 2, we used a factorial ANOVA model to assess the main effects of severity, group, and their interaction on the progression rate. None of these comparisons were statistically significant (p > 0.5 for all effects), indicating that the different distribution of severity did not significantly impact the outcomes between groups.

3.	Implicit control of severity in the MD delta calculation:

Figure 3 explores the association between the delta MD (final – baseline value) and follow-up duration. Because the delta inherently incorporates the baseline MD value, this metric serves as an outcome variable already adjusted for baseline severity. Regression coefficients did not differ significantly between groups, reinforcing the comparability between them.

4.	Additional analysis using another functional index (VFI):

We restructured the analysis in Figure 4 (ΔVFI vs. time) using models stratified by severity. Separate linear regressions were performed for each group (G1 and G2) within each severity level (Initial, Moderate and Advanced Glaucoma), allowing us to compare functional progression directly adjusted for severity. The results remained non-significant in most comparisons, further supporting the robustness of our findings despite baseline heterogeneity (Lines 166 – 168).

Additionally, a new paragraph was added in the discussion, pointing out and discussing this important limitation (Lines 287 – 305).

**Exclusion of patients with IOP < 6 mmHg more than 20% of the time**

Only four eyes were excluded from the initial dataset based on this criterion. Given the minimal number of exclusions (<3% of the total), this step did not materially affect the distribution of eyes between the IOP groups or the representativeness of the sample. This information was included in the manuscript (Line 157).

**Sample Size/Power of the study:**

A post hoc sample size calculation was performed based on the 2×3 factorial ANOVA model (group × severity) used to assess the functional progression rate. A moderate effect size (f = 0.25), an alpha error of 5% (α = 0.05), and a statistical power of 80% (1 – β = 0.80) were considered. The calculation was conducted in Python using the FTestAnovaPower function from the statsmodels library, which indicated that 128 eyes would be required to ensure adequate power. Since the study included 160 eyes, the sample size was considered sufficient for the proposed inferences. Therefore, the exclusion of these few cases did not compromise the statistical power or introduce bias in the comparative analyses.

The sample size calculation was added to the manuscript (Lines 86–91).

**REVIEWER 2:**

**The choice of thresholds (6–9 vs. 10–14 mmHg)**

The suggested revision has been made in the manuscript (Methods, Lines 123 – 129): The upper threshold of 14 mmHg was selected because it is usually accepted as the upper level of the target IOP for advanced glaucoma. The chosen threshold of 6-9 mmHg IOP corresponds to a single-digit IOP, which is often achieved after successful trabeculectomy, while also being outside the range considered hypotonic. A single-digit IOP may be required not only for normal-pressure glaucoma eyes, but also for selected glaucoma patients progressing despite IOP within the target.

**Handling of missing data**

All 160 eyes had baseline and final visual field examinations available for analysis. However, only 73 eyes had intermediate visual field data obtained between 3 and 5 years of follow-up. Analyses were performed using all available data without imputation for missing intermediate tests. Patients with at least baseline and final assessments were included in the longitudinal analysis.

This information was added to the manuscript (Lines 146 – 150).

**The imbalance in baseline MD and glaucoma severity between the two groups**

The baseline imbalance in MD and glaucoma severity between the two groups was acknowledged as a limitation.

However, this potential confounding effect was carefully addressed using multiple complementary analyses, including stratified subanalysis by severity, interaction analysis between group and severity, implicit control of severity through the delta MD calculation, and additional analysis using the VFI functional index. Across all these approaches, results were consistent, indicating that the observed baseline differences did not materially affect the comparative outcomes.

A new paragraph was added in the discussion, pointing out and discussing this important limitation (Lines 287 – 305).

**Lengthy discussion**

The Discussion section has been revised to make it more concise, and detailed descriptions of figures (including lines, slopes, and r values) have been moved to the Results section, as recommended.

**Clinical implications**

Please note that this important aspect was already addressed in the Discussion section of the submitted manuscript. Specifically, we stated that aiming for a more rigorous IOP target in the single digits may be necessary only in isolated cases where progression occurs despite an apparently adequate IOP, and that IOP-independent mechanisms may play a role in many patients. Furthermore, we discussed that maintaining a mean IOP below 15 mmHg after long-term TRAB can provide visual stability regardless of whether the IOP is in the single digits or between 10 –14 mmHg (Lines 331 – 340).

These statements are intended to inform glaucoma specialists that overly aggressive IOP lowering may not be required for all patients, while recognizing that certain subgroups may still benefit from individualized targets.
