# Supplementary material for: Long-term visual field evaluation of trabeculectomy patients with a mean intraocular pressure below 15 mmHg
Source: PLoS One. 2026 Mar 31;21(3):e0345568. doi: 10.1371/journal.pone.0345568 (PMC13037994; doi:10.1371/journal.pone.0345568)
Supplement: Attachment — Submitted filename: 2nd response to reviewers.docx [file pone.0345568.s004.docx]

December 1^st^, 2025

Dear Editor,

We would like to thank you and the reviewers for your time and careful re-evaluation of our manuscript.

Please find below the answers for the points addressed by Reviewer #1:

1. Clarification of “adequate power”

We agree that, because our study uses a retrospective dataset with a fixed sample size, the power calculation is exploratory and cannot be interpreted as a basis for determining the actual sampling. We have revised the Methods section to explicitly state that the power analysis was performed only to assess the sensitivity of the available sample and not to guide recruitment (Lines 94 – 95, 2^nd^ revised manuscript).

2. Potential underestimation of variability in progression

We have added a brief acknowledgment in the Discussion noting that using eyes with fewer visual fields (VFs) may underestimate variability in progression rates, which in turn could affect the stability estimate. This limitation is now explicitly recognized (Lines 154 – 155, 2^nd^ revised manuscript).

3. Condensing the severity-imbalance paragraph

We appreciate the suggestion to improve readability. The paragraph addressing the imbalance in disease severity has been condensed into a shorter, more concise form, without loss of content, to enhance clarity and flow (Lines 272 – 281, 2^nd^ revised manuscript).

Please let us know if you have any further suggestions.

Thank you for the opportunity to be considered for this prestigious journal.

Sincerely,

The authors.
